# The multifaceted actions of the lncRNA H19 in cardiovascular biology and diseases

**DOI:** 10.1042/CS20210994

**Published:** 2022-08-10

**Authors:** Denise Busscher, Reinier A. Boon, Rio P. Juni

**Affiliations:** 1 Department of Physiology, Amsterdam UMC, Vrije Universiteit Amsterdam, De Boelelaan 1117, Amsterdam, the Netherlands; 2 Amsterdam Cardiovascular Sciences, Microcirculation, Amsterdam, the Netherlands; 3 Centre for Molecular Medicine, Institute for Cardiovascular Regeneration, Goethe University Frankfurt am Main, Frankfurt am Main, Germany; 4 German Centre for Cardiovascular Research DZHK, Partner site Frankfurt Rhein/Main, Frankfurt am Main, Germany

**Keywords:** cardiovascular disease, cardiovascular physiology, large intervening non-coding RNA, lncRNA H19, non-coding RNA

## Abstract

Cardiovascular diseases are the leading cause of death and debility worldwide. Various molecular mechanisms have been studied to better understand the development and progression of cardiovascular pathologies with hope to eradicate these diseases. With the advancement of the sequencing technology, it is revealed that the majority of our genome is non-coding. A growing body of literature demonstrates the critical role of long non-coding RNAs (lncRNAs) as epigenetic regulators of gene expression. LncRNAs can regulate cellular biological processes through various distinct molecular mechanisms. The abundance of lncRNAs in the cardiovascular system indicates their significance in cardiovascular physiology and pathology. LncRNA H19, in particular, is a highly evolutionarily conserved lncRNA that is enriched in cardiac and vascular tissue, underlining its importance in maintaining homeostasis of the cardiovascular system. In this review, we discuss the versatile function of H19 in various types of cardiovascular diseases. We highlight the current literature on H19 in the cardiovascular system and demonstrate how dysregulation of H19 induces the development of cardiovascular pathophysiology.

## Introduction

Prolonging of the average human lifespan is presumably one of the greatest achievements to our modern society [1], allowing us to live on average over 81 years across the European Union (EU) [2]. Remarkably, the number of people aged over 85 years or older is projected to expand rapidly from 13.8 million in 2018 to 31.8 million in 2050, showing a drastic growth by 130% [3]. However, the rapid expansion of the elderly population gives rise to many socio-economic concerns because the added years to our lifespans are often accompanied by late-life morbidities [3,4]. In fact, aging has emerged as a predominant - and notably an independent - risk factor for the development of a wide spectrum of chronic diseases including cardiovascular diseases (CVDs) [5–7]. CVDs define a broad spectrum of pathological conditions that affect the heart or blood vessels, including sequelae that arise from damaged vasculature in other organs such as the brain, kidney or eye [8]. Although some advances have been made in the prevention, treatment and prognosis of CVDs, it still remains the leading cause of death worldwide, accounting for 17.8 million deaths annually [9]. In the EU alone, CVD-associated mortality represents 37% of overall deaths, which gives rise to an estimated economic burden of 210 € billion per year [10]. This highlights an urgent need to develop novel diagnostic and therapeutic approaches, which address the root cause of CVDs in order to reduce mortality and improve the quality of life of these patients. Thus, it is of vital importance to understand the underlying cellular mechanisms that drive the development and progression of CVDs in order to combat dysfunctions of the cardiovascular system.

## Long non-coding RNAs

With the advancement of next generation sequencing technologies, it is nowadays well known that a major part of the human genome, initially considered to be ‘junk DNA’ [11], is pervasively transcribed into RNA, while less than 2% of the entire genome is translated into proteins [12–14]. The large number of transcripts that do not encode for proteins are collectively referred to as non-coding RNAs (ncRNA). Based on the threshold of 200 nucleotides in length, ncRNAs can be further divided into two major classes: small ncRNAs or long ncRNAs (lncRNAs). The latter constitutes the largest and functionally most diverse subclass of the non-coding transcriptome [15–18]. Similar to protein-coding transcripts, many lncRNAs are post-transcriptionally modified via alternative splicing, 5′-capping and polyadenylation [19]. In addition, some lncRNAs arise from backsplicing events of linear mRNA to generate more stable circular RNAs in which the 5′ and 3′ ends are covalently closed [20]. In general, lncRNAs show a lower interspecies conservation in their primary sequence, although they may display a higher degree of structural conservation among different species [21]. Moreover, lncRNAs are typically less abundant than mRNAs but exhibit stronger tissue-specific expression patterns [19].

**Figure 1:**
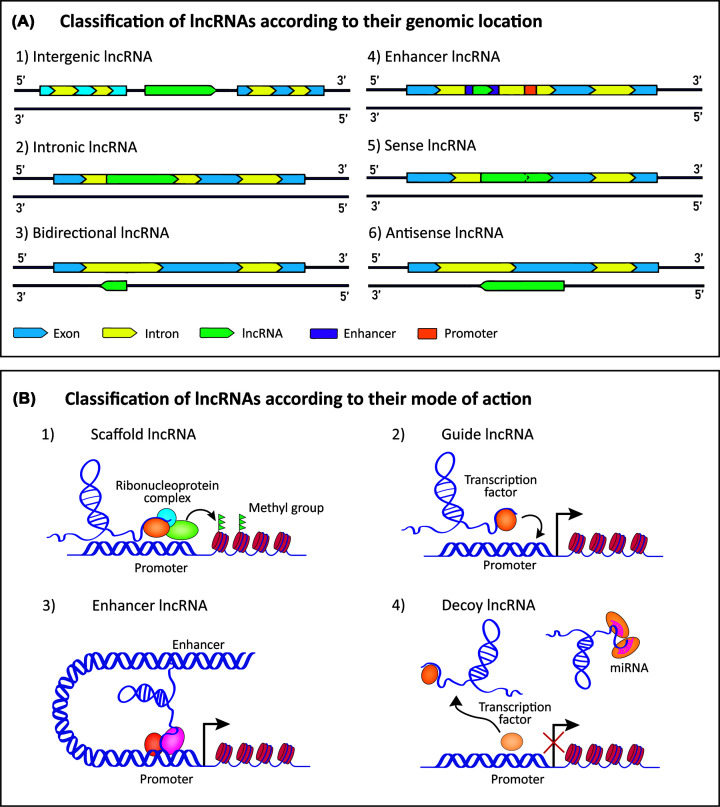
LncRNA classification according to their genomic location (A) or mode of action (B) (**A**) (A1) Intergenic lncRNAs are located between two protein-coding genes, (A2) intronic lncRNAs are located within one of the introns of a protein-coding gene, (A3) bidirectional lncRNAs are situated on the opposite strand of the protein-coding gene, within 1 kb of the promoter of this gene, and are transcribed in the opposite direction of this promoter, (A4) enhancer lncRNAs are located in the enhancer regions of the protein-coding gene, (A5) sense lncRNAs sit within the same strand as the protein-coding gene, can span multiple introns or exons and are transcribed in the same direction as the coding gene, (A6) antisense lncRNAs are located on and transcribed from the opposite strand of a protein-coding gene. (**B**) (B1) LncRNA can act as scaffold linking different proteins, such as different subunits of chromatin modifying complex, required for a synchronized action, (B2) LncRNA can recruit or guide proteins, such as transcription factors, to a specific site on the DNA, (B3) enhancer lncRNAs can link enhancer and promoter regions of genes through chromosomal looping, and (B4) decoy lncRNAs can lead astray the target proteins from DNA or can bind to and compete with miRNAs.

Until now, the NONCODE database estimates over 150,000 lncRNA transcripts to be present in humans and their molecular functions are just beginning to emerge [22]. Consequently, many attempts have been made in order to classify this heterogeneous group of lncRNAs [23,24]. Because a large number of lncRNAs have been shown to impact the expression of their adjacent genes, the first established classification was based on the genomic location, thereby dividing them into six distinct categories: (i) intergenic, (ii) intronic, (iii) bidirectional, (iv) enhancer, (v) sense, or (vi) antisense lncRNAs [25] (Figure 1A). However, lncRNAs regulate not only local chromatin structure and gene expression in *cis* (regulation of target genes that are located at or near the same genomic locus) but also leave the site of transcription to perform a diverse set of cellular functions in *trans* (regulation of target genes at independent genomic loci) [26]. Due to their ability to interact with RNA, DNA, and/or protein, it is now well-known that lncRNAs act on multiple levels of gene regulation, depending in part on their subcellular location. To name a few, lncRNAs have important roles in chromatin remodeling and transcriptional regulation, alternative splicing, organization of nuclear sub-compartments, miRNA regulation, translational regulation, as well as cell-to-cell communication [27,28].

In order to account for the increasing complexity in their mode of action, an alternative classification of lncRNAs was proposed [25,29,30], which takes their targeting mechanisms into account. According to this model, the mechanisms of action by which lncRNAs exert their function can be divided into at least four different archetypes; (i) scaffold, (ii) guide, (iii) enhancer, and (iv) decoy lncRNAs (Figure 1B). However, it should be noted that individual lncRNAs may employ various mechanisms to perform their cellular function, which are therefore not mutually exclusive and may overlap. Acting as a scaffold, lncRNAs can facilitate the formation of ribonucleoprotein complexes, which may induce transcriptional activation or repression of a target gene. Upon binding to a transcription factor or a chromatin modifier, lncRNAs can also provide guidance to a target promoter, thus, modifying the gene expression of a target gene. By interacting with both, enhancer and promoter regions, this archetype of lncRNAs can form a chromosomal looping, bringing them in close proximity to induce gene expression. In contrast, decoy lncRNAs can sequester, and thereby, inhibit the biological function of various RNA-binding proteins, such as transcription factors, chromatin modifiers, and scaffolding proteins. In addition, some lncRNAs act like sponges to prevent miRNAs from binding to their targets (competing endogenous RNA, ceRNA), suggesting a lncRNA–miRNA–target axis. Besides scaffold, guide, enhancer and decoy lncRNAs, a fifth archetype has been described, referred to as signal lncRNAs. This archetype defines a set of lncRNAs that are present at a particular genomic location in a space and time-specific manner to signal for transcriptional activation or repression (e.g KCNQ1OT1) [25,29] as well as lncRNAs that by itself do not have regulatory function, but its transcription process does (e.g. upperhand) [31,32]. By definition, signal lncRNAs serve as mere indicators of transcriptional activity without a secondary biological function as transcript [29]. Therefore, we will not address signal lncRNAs as a distinct mode of action throughout this paper.

In recent years, a growing body of research has shed light on the functional importance of numerous lncRNAs in many biological and pathological processes with particular emphasis of the cardiovascular system [33–35]. Thus, the dynamic regulation of lncRNAs upon initiation and progression of many CVDs highlights their potential to serve as diagnostic biomarkers or as targets for novel therapeutic approaches [25,36].

## The multifaceted role of H19 in cardiovascular disease

**Figure 2:**
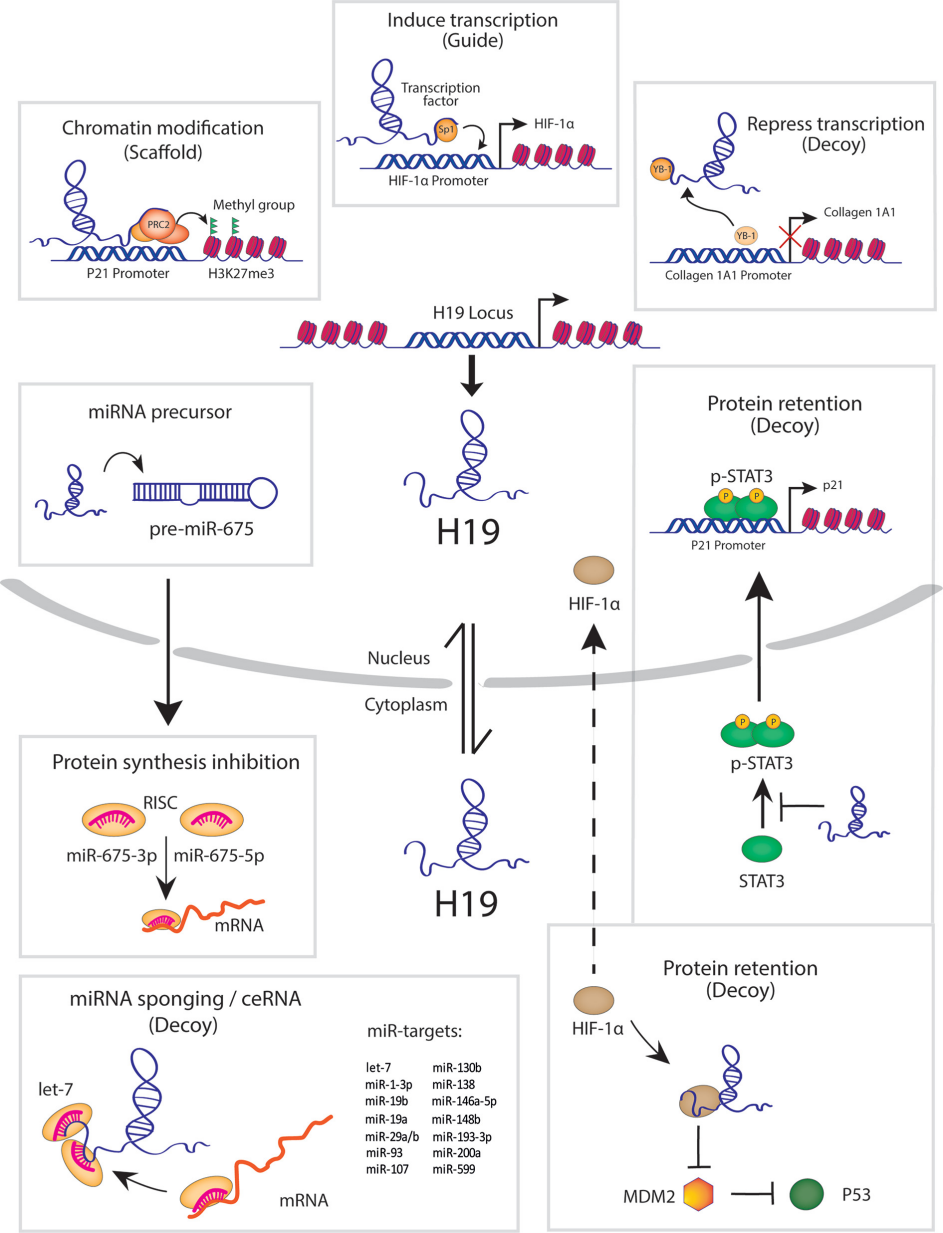
The diverse mode of actions of the lncRNA H19 in cardiovascular diseases H19 can act on multiple levels of gene regulation to exert its biological function. In the nucleus, H19 can serve as a scaffold for PRC2 to promote repressive H3K27me3 on p21 locus. H19 can guide Sp1 to induce HIF1-α expression. Acting as a decoy, H19 sequesters YB-1 to repress collagen 1A1 expression. As a host gene of miR-675, it can repress several target mRNAs from protein synthesis. In the cytoplasm, H19 can interact with HIF1-α to repress its nuclear translocation, thereby promoting p53 stabilization via inhibition of MDM2. H19 acts on STAT3 signaling to repress its nuclear translocation and subsequent p21 induction. Further, H19 can act as a competing endogenous RNA (ceRNA) by sponging several miRNA targets such as let-7. Abbreviations: H3K27me3, H3K27 trimethylation, PRC2, polycomb repressive complex 2, RISC, RNA-induced silencing complex.

As mentioned above, lncRNAs may use multiple strategies in combination to exert their biological function. In particular, the complexity that arises through the multifaceted action of a single transcript can be exemplified by the lncRNA H19 (Figure 2 and Table 1). H19 was one of the first lncRNAs to be discovered, which soon after became an intriguing research focus due to its pivotal role in embryonic development and tumorigenesis [37–40]. The H19 gene is located close to the telomeric region of human chromosome 11p15.5 within a unique cluster of genes that are subjected to genomic imprinting, well-known as the H19/IGF-2 locus. While its neighboring gene IGF (insulin­-like growth factor)-2 is paternally expressed [41], H19 is expressed from the maternal allele and is paternally imprinted [42]. H19 is transcribed by RNA polymerase II into a 2.3 kb spliced, 5′-capped, polyadenylated transcript and is evolutionarily highly conserved [43]. The expression of H19 is significantly up-regulated during embryogenesis but decreases soon after birth [44,45]. In adults, H19 expression is globally repressed, although some tissues, such as the cardiac and skeletal muscle, show a significant basal expression of H19 [46,47]. An increasing number of studies has revealed the implication of H19 in many physiological and pathophysiological processes [38,48–50]. However, the understanding on the mode of action of H19 in cardiovascular dysfunction is still at its infancy. The aim of this manuscript is to summarize the recent findings on H19 in the cardiovascular system and to highlight the impact of its dysregulation in cardiovascular diseases.

**Table 1:** Implications of the lncRNA H19 in various cardiovascular pathologies: dissecting its mode of action, related function and main target mechanisms in the respective *in vitro*/*in vivo* models

H19	Mode of action	Effect of H19 dysregulation	Main target	Model	Ref.
**Diabetic retinopathy**					
↓	Unknown	Promotes EndoMT	TGF-β1/ERK1/2	Vitreous humor of diabetic patients with proliferative DR, high glucose-stimulated human retinal ECs	[51]
↓	ceRNA	Promotes inflammation	miR-19b/SIRT1	High glucose-stimulated ARPE-19 cells	[52]
↓	ceRNA	Promotes inflammation	miR-93/XBP1	High glucose-stimulated ARPE-19 cells	[53]
→	Unknown	Unknown	Unknown	Serum of diabetic patients with and without DR	[54]
**Atherosclerosis**					
↑	Unknown	Promotes VSMC proliferation and inhibits apoptosis	ACP5	Serum of patients with atherosclerosis	[55]
↑	ceRNA	Promotes VSMC proliferation, migration, and invasion	miR-599/PAPPA	Serum of patients with atherosclerosis, HFD-treated ApoE^−/−^ mice, and ox-LDL-treated human aortic VSMC	[56]
↑	ceRNA	Promotes lipid accumulation and inflammatory response	miR-130b	Serum of patients with atherosclerosis, ox-LDL-treated Raw264.7 macrophages	[57]
↑	ceRNA	Promotes VSMC proliferation and inhibits apoptosis	miR-148b/Wnt/β-catenin	Serum of atherosclerotic patients, ox-LDL-stimulated human aortic VSMCs	[58]
↑	ceRNA	Promotes proliferation and suppresses apoptosis	miR-19a/p38, p65/NF-κB	Serum of patients with atherosclerosis, atherosclerotic plaques of ApoE^−/−^mice	[59,60]
↑	ceRNA	Promotes inflammation, apoptosis, and oxidative stress	Let-7/periostin	Serum of patients with atherosclerosis, ox-LDL-treated HUVECs	[61]
↑	Unknown	Unknown	Unknown	Coronary artery plaque of patients with atherosclerosis, developing rabbit thoracic aorta	[62]
↑	ceRNA	Promotes lipid accumulation in macrophages	miR-146a-5p/ANGPTL4	Blood and aortic root of ApoE^−/−^mice fed with HFD	[63]
↑	Unknown	Promotes proliferation and inhibits apoptosis of VSMCs	p53/BAX	Atherosclerotic aorta of HFD-treated ApoE^−/−^mice	[64]
↑	Guide (transcriptional repression)	Promotes vulnerable plaque formation and intraplaque angiogenesis	CTCF/PKD1	Aortic tissues of atherosclerotic ApoE^−/−^mice on HFD	[65]
↑	ceRNA	Promotes TGF-β-signaling and induces EndoMT	Let-7/TET1	ECs of human atherosclerotic coronary arteries, streptozotocin-induced mouse pulmonary microvascular ECs, TNF-α-stimulated HUVECs, and HAoECs	[66]
↓	Unknown (inhibition of STAT3 activation)	Promotes EC senescence and inflammatory activation, inhibits proliferation, and angiogenic sprouting	STAT3/IL-6	ECs of human atherosclerotic plaques compared with ECs of healthy arteries	[67]
↓	ceRNA	Promotes EndoMT	miR-148b-3p / ELF5	Ox-LDL-treated HUVECs	[68]
**Cardiac disease**					
↑	Unknown	Unknown	Unknown	Serum of patients with CAD	[69–72]
→	–	–	–	ECs and circulating small extracellular vesicles from plasma of patients with CAD	[73]
↑	ceRNA	Promotes EndoMT	Let-7/TET1	Human coronary arteries from patients with mild, moderate or severe CAD	[66]
↑	Decoy	Promotes cardiac fibrosis and cardiac remodeling	YB-1/Collagen 1A1	Infarct border zone in a mouse model of LAD ligation	[74]
↑	Unknown	Inhibits cardiac inflammation, apoptosis, and hypertrophic cardiac remodeling	Vitamin D receptor	Infarcted zone in a mouse model of LAD ligation	[75]
↓	Unknown	Modulates autophagy	Beclin-1, ATG7, and LC3 II/I ratio	Infarct region of a LAD ligation mouse model	[76]
↓	Unknown	Unknown	Unknown	Cardiac tissue of a LAD ligation rat model	[77]
↓	ceRNA	Induces CM apoptosis	miR-877-3p/BCL-2, BAX	Hearts of a I/R mouse model, H_2_O_2_-treated CMs	[78]
↑	Decoy	Promotes hypertrophy	PRC2 / tescalcin / NFAT	Initial phase (2–4 weeks) in TAC-induced mouse model of cardiac hypertrophy and HF	[79]
↓				Decompensated phase (4–6 weeks) in TAC-induced mouse model of cardiac hypertrophy and HF, pathological human heart samples, hypertrophic pig hearts, isoproterenol-treated hiPSC-derived CMs, human engineered heart tissue upon afterload enhancement	[79]
↑	miRNA- precursor	Increases CM apoptosis	miR-675/PA2G4	Myocardial tissue from adriamycin-induced rat model of dilated cardiomyopathy	[80]
↑	miRNA- precursor	Inhibits CM hypertrophy	miR-675/CaMKIIδ	Human HF samples, TAC-induced mouse model of cardiac hypertrophy, and phenylephrine-treated CMs	[81]
↓	ceRNA	Induces apoptosis and inflammation	miR-29a/IGF-1	Cardiac tissue from HFD-induced mouse model of myocardial injury	[82]
**Cerebrovascular disease**					
↑	Scaffold	Promotes neuroinflammation, M1 microglial polarization	HDAC1	Serum of patients with ischemic stroke, as well as plasma, white blood cells, and ischemic brain tissue of MCAO mice	[83]
↑	Unknown	Induces excessive autophagy	DUSP5/ERK1/2	MCAO rat model and OGD/R-treated SH-SY5Y cells	[84]
↑	Unknown	Inhibits neurogenesis	p53/Notch1	Serum of patients with ischemic stroke, MCAO mouse model	[85]
↑	Unknown	Promotes axon sprouting	IGF1R, pS6/mTOR	Sensorimotor cortex of MCAO mouse model	[86]
↑	ceRNA	Regulation of NLRP3/6 inflammasome balance, induces microglial pyroptosis, cytokines overproduction, and neuronal death	miR-21/PDCD4	Retinal I/R mouse model, OGD/R model with primary retinal microglia, and ganglion cells	[87]
↑	ceRNA	Induces oxidative stress and apoptosis	miR-19a-3p/PTEN/AKT3	MCAO mouse model and OGD/R-treated SH-SY5Y cells	[88]
↑	ceRNA	Induces apoptosis and inflammatory response	miR-29b/SIRT1/PGC1α	MCAO mouse model and OGD/R-treated HT22 cells	[89]
↑	ceRNA	Induces oxidative stress and neural apoptosis	miR-148-3p/Rock2	MCAO mouse model and OGD/R-treated N2a cells	[90]
↑	Scaffold, miRNA- precursor, miRNA biogenesis	Induces NSC differentiation, mediator of stroke-induced neurogenesis	PRC2/p21, PTEN, p27, miR675/TGF-β1, Dicer	NSCs of the subventricular zone of rats subjected to focal cerebral ischemia	[91]
↑	ceRNA	Blocks excessive autophagy	miR-29b/AKT3/mTOR	Serum of human newborns with HIE, brain tissue of neonatal HIE rats	[92]
↓	ceRNA	Induces neuronal apoptosis	miR-107/VEGF	HIBD rat models established by partial occlusion of carotid artery	[93]
↓	ceRNA	Induces NCS apoptosis	miR-107/Wnt/β-catenin/PI3K/AKT3	Hypoxia-treated NSCs	[94]
↑	Unknown	Unknown	Unknown	Most up-regulated lncRNA from striatum of 2 ICH rat models (collagenase or blood injection)	[95]
↓	miRNA-precursor / ceRNA	Promotes neuronal apoptosis	miR-675/p53, let-7a/NGF	Brain cortex of SAH mouse model (endovascular perforation)	[96]
↓	miRNA-precursor / ceRNA	Unknown	miR-675/HIF-1α miR-138/eNOS	Brain of SAH mouse model (endovascular perforation)	[97]
**Aneurysmal disease**					
↑	Unknown	Unknown	Unknown	Most up-regulated gene in human intracranial aneurysm tissue	[98]
↑	Guide, Decoy	Promotes SMC apoptosis and inhibits SMC proliferation	Sp1/HIF-1α, HIF-1α/p53	Human AAA tissue, LDLR(-/-) Yucatan mini-pig aneurysm model, Ang II-infused ApoE^−/−^ mice	[99]
↑	ceRNA	Promotes vascular inflammation	Let-7/IL-6	Human AAA samples and Ang II-perfused ApoE^−/−^mouse aortas, Ang II-treated VSMCs and macrophages	[100]
↑	ceRNA	Promotes VSMC apoptosis and inhibits VSMC proliferation, induces ECM degradation	miR-1-3p/ADAM10	Human TAA tissue, serum, and VSMC	[101]
↑	ceRNA	Promotes proliferation and migration, induces phenotypic switch of VSMC (from contractile to synthetic)	miR-193-3p/MMP-2 and MMP-9	Thoracic aorta tissues of patients with aortic dissection, Ang II-infused ApoE^−/−^mice	[102]
**Pulmonary arterial hypertension**					
↑	miRNA-precursor	Induces CM hypertrophy and fibrosis	miR-675/E2F1/EZH2	Serum and RV from PAH patients, lungs and RV from 2 PAH rat models (MCT injection and pulmonary artery banding)	[103]
↑	ceRNA	Promotes PASMC proliferation	let-7b/AT1R	Serum and lungs of 2 MCT-induced PAH rodent models (SD rats, C57BL/6 mice), cytokine-stimulated PASMCs	[104]
↓	ceRNA	Inhibits apoptosis and induces proliferation of PASMCs	miR-675-3p/IGF1R, miR-200a/PDCD4	PASMC of MCT-induced PAH rat model	[105]

Abbreviations: AAA, abdominal aortic aneurysm; Ang II, angiotensin II; ApoE^−/−^, apolipoprotein E-deficient; CAD, coronary artery disease; ceRNA, competing endogenous RNA; CM, cardiomyocyte; DR, diabetic retinopathy; EC, endothelial cell; EndoMT, endothelial-to-mesenchymal transition; eNOS, endothelial nitric oxide synthase; HAoEC, human aortic EC; hiPSC-derived CM, human induced pluripotent stem cell-derived CM; HIBD, hypoxic-ischemic brain damage; HIE, hypoxic–ischemic encephalopathy; HFD, high-fat diet; HUVEC, human umbilical vein EC; ICH, intracerebral hemorrhage; I/R, ischemia reperfusion; LAD, left anterior descending artery; MCAO, middle cerebral artery occlusion; MCT, monocrotaline; NSC, neuronal stem cell; OGD/R, oxygen-glucose deprivation/re-oxygenation; oxLDL, oxidized low-density lipoprotein; PAH, pulmonary arterial hypertension; PASMC, pulmonary arterial smooth muscle cell; RV, right ventricle; SAH, subarachnoid hemorrhage; Sp1, specificity factor 1; TAC, transverse aortic constriction; VSMC, vascular smooth muscle cell.

### H19 in diabetes mellitus-induced retinopathy

**Figure 3:**
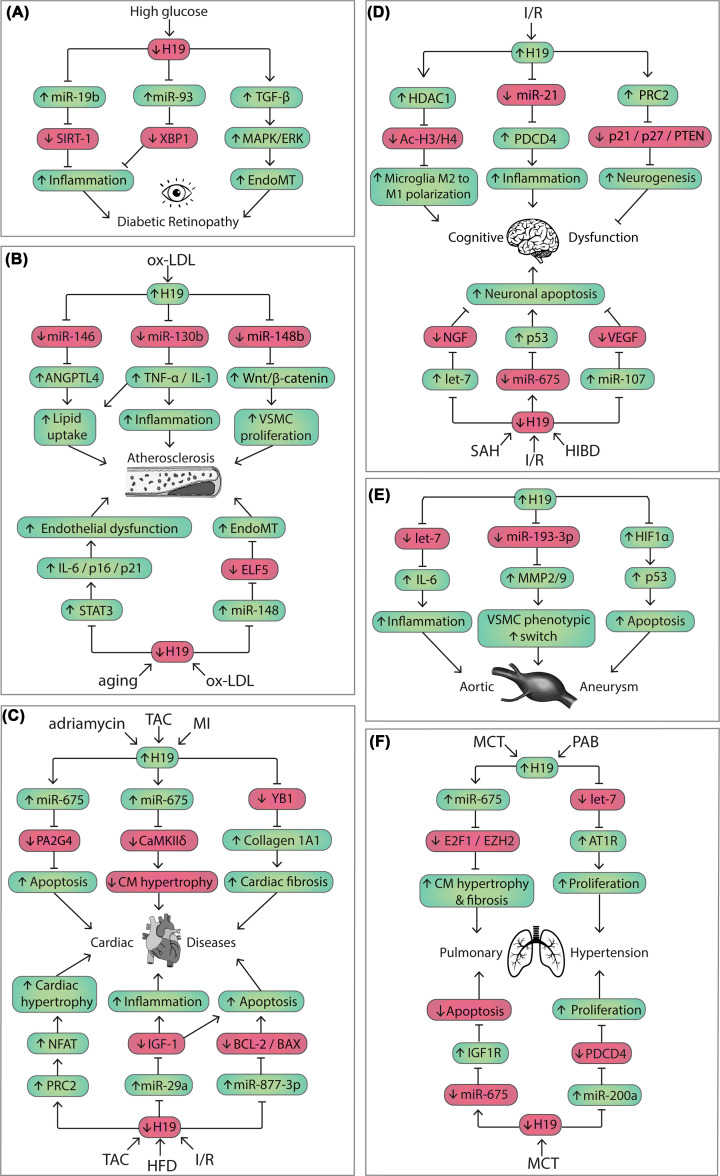
Molecular mechanisms of the lncRNA H19 in various cardiovascular pathologies, focusing on (A) diabetes mellitus-induced retinopathy, (B) atherosclerosis, (C) cardiac disease, (D) cerebrovascular disease, (E) aneurysmal disease, and (F) pulmonary hypertension Abbreviations: CM, cardiomyocyte; EndoMT, endothelial-to-mesenchymal transition; HIBD, hypoxic-ischemic brain damage; HFD, high-fat diet; MI, myocardial infarction; I/R, ischemia–reperfusion; MCT, monocrotaline; ox-LDL, oxidized low-density lipoprotein; PAB, pulmonary artery banding; SAH, subarachnoid hemorrhage; TAC, transverse aortic constriction; VSMC, vascular smooth muscle cell.

Diabetic retinopathy (DR) is a frequent secondary complication in patients with type 1 and 2 diabetes and represents a leading cause of visual impairment and blindness in the middle-aged and elderly population [51–106]. Accumulating evidence suggests an underlying chronic inflammation during the pathogenesis of DR. Numerous proinflammatory cytokines, chemokines, and growth factors are up-regulated in the vitreous or aqueous humor of diabetic patients with DR, including tumor necrosis factor (TNF)-α, interleukin (IL)-1β, and IL-6 [107]. H19 appears to be central in modulating proinflammatory processes that underlie the development of DR. Under high-glucose conditions, H19 expression levels were shown to be down-regulated in human retinal endothelial cells [51] and ARPE-19 retinal epithelial cells [52,53]. Hyperglycemia-induced reduction of H19 expression was confirmed in the vitreous humor from diabetic patients with proliferative DR as compared with non-diabetic controls [51]. Mechanistically, H19 modulates high glucose-induced proinflammatory cytokine production in ARPE-19 cells via X-box-binding protein 1 (XBP1), a transcription factor that is targeted by miR-93. Using dual-luciferase reporter and RNA immunoprecipitation (RIP) assays, it was demonstrated that miR-93 is a direct binding target for H19 (Figure 3A). Overexpression of H19 leads to an increased XBP1 expression by sponging of miR-93, resulting in a significant reduction of inflammatory cytokines including TNF-α, IL-1β, and IL-6 [53]. In addition to its influence on the miR-93/XBP1 axis, H19 can rescue the high glucose-induced inflammatory response via the miR-19b/sirtuin-1 (SIRT1) axis [52]. H19 can directly bind to miR-19b, leading to an increased SIRT1 expression in ARPE-19 cells and reduced expression of TNF-α, IL-1β, and IL-6 (Figure 3A) [52].

Further, down-regulation of H19 in the retinal endothelium causes phenotypic switching through up-regulation of transforming growth factor (TGF)-β1 and the subsequent cellular process known as endothelial-to-mesenchymal transition (EndoMT) [108,109]. Using an H19 knockout mouse model, the authors reported a similar diabetes-like EndoMT phenotype in the retina with decreased endothelial markers CD31 and vascular endothelial (VE)-cadherin as well as increased mesenchymal markers fibroblast-specific protein 1 (FSP-1), transgelin/SM22, α-smooth muscle actin (α-SMA), and vimentin. Conversely, overexpression of H19 leads to a reduction in TGF-β1 levels and can thereby prevent the hyperglycemia-induced EndoMT by blocking the TGF-β-mediated activation of the mitogen-activated protein kinase (MAPK) pathway via extracellular-signal-regulated kinase (ERK) 1/2 (Figure 3A) [51]. Overall, H19 plays a central role in the pathogenesis of DR by keeping in check two distinct pathological processes, namely excessive inflammation and EndoMT (Figure 3A). As EndoMT shares similar characteristics as inflammatory response and requires proinflammatory cytokines to be fully activated [108,109], it is plausible that the anti-EndoMT effect of H19 is secondary to its anti-inflammatory effect.

In an attempt to use H19 as diagnostic or prognostic biomarker for early prediction of DR, a more recent study compared H19 expression levels in the circulation of diabetic patients with and without DR [54]. In contrast with the decreased H19 levels in the retinal tissues or the vitreous humor [51–53], circulating H19 was found up-regulated in patients with type 2 diabetes compared with healthy controls [54]. This suggests that circulating H19 can be used as an additional biomarker for type 2 diabetes. However, this increased plasma H19 levels do not necessarily mean that it is promoting diabetes, and therefore detrimental. It is plausible that H19 expression is induced in other tissues as a protective compensatory mechanism against the hyperglycemic insult. In this scenario, H19 might be actively secreted or released to the circulation when there is damage inflicted to the cells [110]. Nevertheless, it was reported that diabetic individuals with or without DR showed no significant differences in plasma H19 levels, and therefore, failed to correlate with DR grades [54].

### H19 in atherosclerosis

Atherosclerosis is characterized by lipid accumulation in the artery intima, which forms the pathological underlying mechanism of various CVDs, such as coronary artery disease (CAD) and heart failure (HF) [111]. In the developing fatty streak and the more advanced atherosclerotic lesions, uptake of oxidized low-density lipoprotein (ox-LDL) by recruited macrophages and vascular smooth muscle cells (VSMCs) results in the formation of foam cells, a process that represents a major hallmark of atherosclerosis [112].

#### Proatherogenic function of H19

##### The role of H19 in macrophages

H19 has been reported to be up-regulated in the serum [55–61] and the coronary artery plaque [62] of patients with atherosclerosis. Nevertheless, the detection of H19 in the plaque was only performed in patients and therefore lacked a healthy control group [62]. In addition, H19 was shown up-regulated in the blood [63] and aortic root [63–65] of atherosclerosis-prone apolipoprotein E-deficient (ApoE^−/−^) mice fed with western diet as compared with the control C57BL/6 mice on chow diet [63]. H19 silencing was reported to reduce plaque accumulation, while its overexpression induces progression of plaque formation [63]. Mechanistically, H19 promotes lipid uptake of ox-LDL-treated THP1 macrophages by acting as a ceRNA to miR-146-5p, leading to the de-repression of its target gene angiopoietin-like 4 (ANGPTL4) (Figure 3B) [63]. Likewise, H19 expression was up-regulated in RAW264.7 macrophages treated with ox-LDL [57]. Knockdown of H19 decreased ox-LDL-induced lipid accumulation and counteracted the up-regulation of triglycerides, total cholesterol and LDL-cholesterol as well as the down-regulation of HDL-cholesterol. Further, H19 knockdown was associated with decreased expression of proinflammatory markers TNF-α and IL-1β in CD68+ macrophages, while increasing the expression of anti-inflammatory markers IL-4 and IL-10 in CD163+ cells. It is suggested that H19 drives the atherosclerosis phenotype in ox-LDL-treated Raw264.7 cells by promoting lipid dysregulation and inflammatory response by targeting miR-130b (Figure 3B) [57].

##### The role of H19 in VSMCs

Another study proposes that H19 induces atherosclerosis by inhibiting apoptosis of VSMCs [55], which promotes foam cell formation and neointimal growth. Although the molecular mechanism was not fully elucidated, it was claimed that H19 asserts this effect by targeting acid phosphatase 5 (ACP5), based on luciferase reporter assay [55]. Further, it was reported that H19 exerts an anti-apoptotic effect via p53. Down-regulation of H19 in VSMCs increases p53 levels and its interaction with B-cell lymphoma (BCL2)-associated X (BAX), leading to increased caspase-3 expression and cell apoptosis [64]. Moreover, it was demonstrated that H19 competitively binds to miR-148b to induce the Wnt/β-catenin signaling pathway in ox-LDL-stimulated human aortic VSMCs, while its depletion suppressed proliferation and induced apoptosis (Figure 3B) [58]. A similar effect was reported where exposure of human aortic VSMCs to ox-LDL induced H19 level, while H19 silencing suppressed ox-LDL-induced VSMC proliferation, migration, and invasion [56]. H19 was suggested to act by sponging miR-599, thereby up-regulating its target pappalysin 1 (PAPPA). Silencing of H19 in ApoE^−/−^ mice fed with high-fat diet (HFD) improved circulating cholesterol, triglyceride, and LDL level, although the plaque size was not assessed [56].

##### The role of H19 in endothelial cells

Another mechanistic study suggests that H19 induces endothelial cell inflammatory activation and EndoMT via let-7 inhibition and TET1 activation [66]. This outcome is in contrast with an above mentioned study in the retinal endothelium, where H19 inhibits EndoMT [51], and our own observation in cardiac microvascular endothelium, where H19 was reduced upon proinflammatory stimulation and H19 silencing induced expression of EndoMT-related gene expression (data not shown). Given that Cao et al. [66] used human umbilical vein endothelial cells (HUVECs) and Thomas et al*.* [51] and our group used retinal and cardiac microvascular endothelium, respectively, this discrepancy may be due to the difference of H19 regulation in endothelial cells from different vascular beds, as it has been shown that there is endothelial heterogeneity depending on their organ of origin [113–115]. While it was reported that H19 was up-regulated in the endothelium upon ox-LDL stimulation [61], it could well be a compensatory mechanism to protect the cells. It has been reported that H19 exerts an anti-apoptotic effect and promotes endothelial cell proliferation, *in vitro*, where it acts as ceRNA for miR-19a, leading to increased expression of p38 and p65, two key factors implicated in the nuclear factor-kappa B (NF-κB) pathway, thus promoting proliferation and suppressing apoptosis [59,60].

#### Atheroprotective function of H19

In contrast to the above-mentioned literature, other studies report an atheroprotective function of H19. One study showed a reduced expression of H19 in human carotid plaques as compared with control arteries from healthy donors [67]. H19 was shown to be very abundant in endothelial cells and the loss of H19 led to an up-regulation of p16 and p21, increased inflammatory markers, reduced proliferation and increased senescence of endothelial cells, all aspects required for atherosclerotic plaque formation [67]. While H19 expression was repressed by aging, overexpression of H19 improved endothelial function in aortic rings of aged mice. Mechanistically, it was demonstrated that H19 inhibits the signal transducer and activator of transcription 3 (STAT3)/IL-6 signaling pathway (Figure 3B) [67]. In an attempt to study the protective effect of Icariin on atherosclerosis, it was further demonstrated that enhanced expression of H19 by Icariin inhibited EndoMT in the endothelium upon ox-LDL treatment, possibly through miR-148b-3p and its target E74-like factor 5 (ELF5) (Figure 3B). H19 knockdown reversed the protective effect of Icariin on endothelial cell function and impaired cell migration as measured by wound-healing and trans-well assays [68].

Collectively, these findings show some discrepancies on the role of H19 in atherosclerosis. There were two evidences on the expression of H19 in human atherosclerotic plaque [62,67], which seem to be conflicting. However, the study showing up-regulation of H19 in the plaques [62] would benefit from including healthy control samples in their analysis to further confirm these findings. The *in vivo* mouse studies incline to show increased levels of H19 in the atherosclerotic plaque [63–65]. The role of H19 in the atherosclerotic plaque appears to be cell type-specific. In the endothelium, H19 promotes cell viability and proliferation, and prevents apoptosis [67]. In other cells, such as VSMCs and macrophages, which are considered the more pre-dominant cell type in atherosclerotic plaques [116], H19 displays different functions. In VSMCs, H19 seems to prevent apoptosis and induce a phenotypic switch to a synthetic form, where VSMCs are more active in proliferation and migration [117]. In macrophages, it appears to promote polarization toward a proinflammatory phenotype in order to clear up oxidized lipid particles and repair the damage in the vessel wall [83]. However, these supposedly protective effects become detrimental in the long term, as VSMCs and macrophages accumulate more lipids and transform into foam cells, which in turn contribute to the progression of atherosclerotic plaques. Whether or not silencing or overexpression of H19 is therapeutic, it may depend on the target cells and the timing of administration. The systemic administration may be difficult to differentiate the target cell, although there is some evidence that a certain dose of silencing agents may target only endothelial cells lining the vessel wall, while higher doses can target the parenchymal cells as well [118,119]. The timing is particularly crucial as silencing at the early stage might be detrimental while at the later stage, where there is excessive and prolonged proliferative and proinflammatory response, it may be proven beneficial.

### H19 in cardiac pathologies

#### H19 dysregulation in coronary artery disease (CAD)

H19 has been shown to be involved in cardiac pathologies, such as CAD, ischemia/reperfusion (I/R) injury, and myocardial infarction (MI). CAD refers to the pathological process, obstructive or non-obstructive, affecting the coronary arteries [120]. The level of H19 in the circulation was reported to increase in patients with CAD from a Chinese population [69–71]. However, a study on an Iranian population [72] showed no significance compared with the healthy control group. In addition, a study analyzing lncRNA expression in circulating small extracellular vesicles (sEVs) from control and CAD patients found no up-regulation of H19 in patients with CAD as compared with control [73]. Interestingly, single-nucleotide polymorphisms (SNPs) in the H19 gene has been associated with decreased susceptibility to CAD in a Chinese population [71,121], suggesting a protective effect of these H19 SNPs. Given the heterogeneity in the results of different patient populations, the association between H19 plasma levels and CAD needs to be reevaluated in multicenter multiethnic studies to provide a more robust conclusion on whether increased H19 levels are positively or negatively associated with the occurrence of CAD.

#### H19 dysregulation upon myocardial infarction (MI)

With regards to MI, increased tissue levels of H19 have been negatively and positively associated with increased infarct size. It was shown that the expression of H19 increased at the infarct area 4 days post MI in a mouse model of left anterior descending (LAD) artery ligation [74]. Cardiac overexpression of H19 resulted in severe cardiac dilation and fibrosis with up-regulation of several extracellular matrix genes, while genetic ablation of H19 by CRISPR-Cas9 ameliorated post-MI cardiac remodeling. Y-box-binding protein (YB)-1, a suppressor of collagen 1A1, was identified as an interacting protein of H19. It was proposed that H19 acted to antagonize YB-1 through direct interaction under hypoxia, resulted in de-repression of collagen 1A1 expression and cardiac fibrosis (Figure 3C) [74]. Interestingly, another study reported that increased H19 expression at the infarcted zone started 24 h following permanent LAD occlusion and reached the highest concentration at day 7, which then decreased there after [75]. However, this up-regulation of endogenous H19 after MI appears to be cardioprotective, since the global knockout of H19 exaggerated cardiac inflammation and hypertrophic cardiac remodeling [75]. In line with this putative cardioprotective effect of H19, reduced expression of H19 has also been observed at the later stage of 3 weeks post-MI in the infarct region of a LAD ligation mouse model, whereas overexpression of H19 with lentivirus pcDNA-H19 reduced infarct size and improved cardiac function [76]. Mechanistically, H19 overexpression increased the expression of Beclin-1, ATG7, and LC3 II/I ratio, thereby promoting physiological autophagy response [76]. Further, H19 levels are reduced in rat cardiac tissue 6 weeks post LAD ligation and were rescued to normal levels after exercise, which is associated with reduced fibrosis and improved heart function [77]. Likewise, decreased H19 expression was observed in the heart of an I/R mouse model, while its overexpression improved the outcome of myocardial I/R injury and rescued cardiomyocytes (CMs) from apoptosis [78]. By targeting miR-877-3p, H19 modulates BCL-2/BAX ratio in favor of survival, thereby preventing cytochrome c release and activation of caspase-3 and caspase-9 in mice facing myocardial I/R injury (Figure 3C) [78].

Overall, these studies show a time-dependent expression of H19 upon different types of ischemic insults. Moreover, there is a component of cell type-specific expression of H19 playing a role in the progression MI. In the permanent LAD model, whether it is in mouse or rat, H19 levels increase acutely shortly after injury. It is plausible that this up-regulation is an acute compensatory mechanism of the heart to limit the damage. Increased H19 expression in the first week of MI was shown to occur predominantly in cardiac fibroblasts, which led to induction of cardiac fibrosis [74], possibly as a compensatory mechanism to limit infarct spreading and eventual cardiac rupture. Interestingly, despite using AAV9 as delivery vector intravenously, H19 overexpression was also shown to accumulate predominantly in cardiac fibroblasts, leading to enhanced cardiac fibrosis, plausibly by promoting fibroblast switch to myofibroblast [74]. In addition, it appears that the time line of H19 up-regulation [74] overlaps with the inflammatory response upon MI [122], suggesting that H19 may also be up-regulated in immune cells in the heart, which are predominantly cardiac resident macrophages [123]. This may be intended to activate the cells [83] in order to absorb the necrotic tissue. However, overexpression of H19 in these cells may cause overactivation and an excessive inflammatory response, leading to further tissue damage.

Upon long exposure to ischemia, however, this compensatory mechanism fails as H19 expression decreases, leading to deterioration of cardiac function. In I/R injury, H19 levels decrease shortly after reperfusion [78]. Restoration of H19 expression by direct injection of the overexpression vector in the ventricle at this point displayed a beneficial effect [76,78]. This method of delivery seems to bring H19 overall in the cardiac tissue [124]. Thus, H19 appears to be protective in both, the early and later phase of MI. Nevertheless, timing and cell-specific targeting for H19 overexpression is crucial to harness the positive effect. Up-regulation in cardiac fibroblasts or macrophages at the early phase can be detrimental. However, gain of function at the late stage, when its cardiac expression is already reduced, may be proven therapeutic.

#### H19 as key player in cardiac remodeling

Various insults such as MI or I/R injury, chemotherapy and pressure overload, may lead to further cardiac remodeling that can eventually develop into HF. H19 has been shown to be enriched in the heart [79]. The abundant postnatal cardiac expression of H19 is particularly due to its enrichment in cardiac endothelial cells [79], which have been shown to regulate CM function and are affected in the progression of cardiac remodeling [118,125,126]. Nevertheless, the role of H19 in cardiac remodeling has been mostly focused on CMs. In a rat model of adriamycin-induced dilated cardiomyopathy, H19 expression was increased and its suppression attenuated CM apoptosis and improved left ventricular function [80]. It was shown that adriamycin induced the expression of H19 and miR-675, which then inhibited an anti-apoptotic gene PA2G4, triggering apoptosis of CMs (Figure 3C) [80]. Another study showed that H19 expression was up-regulated in a mouse model of pathological cardiac hypertrophy and HF induced by transverse aortic constriction (TAC) [81]. Interestingly and in contrast with the previous study [80], H19 overexpression in CMs mitigated cell hypertrophic response, whereas its silencing increased CM size. Here, H19 also acted as a host gene for miR-675, which displays the same expression pattern as H19 and was demonstrated to repress its downstream target prohypertrophic gene CaMKIIδ, leading to attenuation of cardiac hypertrophy and improved function (Figure 3C) [81].

This is in line with another study showing increased H19 expression 2 weeks post pressure overload induced by TAC, which was later down-regulated during the progression to the decompensated stage of HF at 4 weeks onwards [79]. Here, however, miR-675 expression did not seem to follow the expression pattern of H19 and remained relatively constant. Moreover, repression of H19 was observed in the heart of a pig model of cardiac hypertrophy and in human tissue. Prohypertrophic stimulation with isoproterenol reduced the expression of H19 in human induced pluripotent stem cell (iPSC)-derived CMs. Likewise, H19 expression was down-regulated in human engineered heart tissue upon afterload enhancement, as well as in various diseased human heart samples from patients with aortic stenosis, hypertrophic cardiomyopathy, and HF. Intriguingly, cardiac hypertrophy was augmented in H19 knockout mice following TAC as compared with wild-type littermates, and cardiac-specific overexpression of H19 via vector-based gene therapy ameliorated cardiac dysfunction of TAC-operated mice. Of note, therapeutic overexpression of the human H19 homolog (instead of murine H19) was similarly effective in blocking cardiac hypertrophy of TAC-operated mice, highlighting again the strong translational potential of H19 in the heart. H19 exerted its anti-hypertrophic function by binding to the polycomb repressive complex 2 (PRC2) to prevent PRC2-mediated epigenetic repression of the tescalcin locus [79], a negative regulator of the prohypertrophic nuclear factor of activated T-cells (NFAT) [127], leading to reduced NFAT expression and activity and improved cardiac function (Figure 3C) [79]. Similarly, H19 was shown down-regulated after 8 weeks of HFD and was reported to mediate the cardioprotective effect of ghrelin in this HFD-induced HF mouse model and in an *in vitro* model of palmitic acid-induced CM hypertrophy. H19 deletion abrogated the effects of ghrelin against apoptosis and inflammation. Mechanistically, H19 was shown to act on miR-29a to repress its inhibition on IGF-1, which then inhibits apoptosis and inflammation (Figure 3C) [82].

Overall, pressure overload appears to induce a similar H19 expression pattern in the heart as ischemic insult, with an acute compensatory increase of H19 levels shortly after injury, followed by reduced expression at the later time point [79], as a sign of a failing compensatory mechanism. While 8 weeks HFD also suppressed H19 levels [82], adriamycin-induced dilated cardiomyopathy seems to give a different response, where increased expression of H19 was observed after 3 weeks and its down-regulation attenuated CM apoptosis and improved left ventricular function [80]. These studies show that different insults can lead to different H19 expression patterns. Pressure overload and HFD are well known to induce cardiac hypertrophy, while the doxorubicin model of cardiomyopathy seems to cause CM apoptosis and heart atrophy. This suggests that with different forms of cardiomyopathies, there might be several distinct downstream pathways leading to differential H19 expression and its specific function. The underlying mechanism is unclear, however, it may again be due to the cardiac cell type-specific function of H19. Future studies investigating the differential expression of H19 in various cell types of the heart upon hypertrophic or carcinogenic stimuli and upon overexpression strategies are needed to decipher these pathways.

### H19 in cerebrovascular diseases

With increasing age, progressive vascular remodelling and atherosclerosis can lead to the development of cerebrovascular diseases, which define a variety of conditions or disorders that affect the blood vessels or disturb the blood supply in the brain, including cerebral small vessel disease [128], vascular dementia [129], and stroke [130]. As second leading cause of death worldwide, the latter accounts for 5.5 million deaths per year [131], and can be classified into two major categories, ischemic stroke (IS) and hemorrhagic stroke (HS). Both conditions are characterized by a lack of oxygen and nutrient supply to the brain. Tissue hypoxia triggers a complex molecular cascade resulting in oxidative stress, excitotoxity, inflammation and breakdown of the blood–brain barrier [132].

#### H19 polymorphisms and circulating levels are associated with IS

Analysis of SNPs in the H19 gene revealed an increased risk for IS in patients that harbor a C to T variation of rs217727 or a C to A variation of rs4929984 [84]. Likewise, rs217727 polymorphism in the H19 gene has been associated with susceptibility to IS in a northern Chinese Han [133] and Iranian population [134]. In contrast, another study conducted in a southern Chinese Han population failed to correlate H19 rs217727 and rs4929984 gene polymorphisms with susceptibility to IS, even though these gene variants are associated with some risk factors for IS, such as diastolic blood pressure, coagulation function and homocysteine levels [135]. This is in line with previous findings from a gene-centric meta-analysis in 68,368 individuals of European ancestry, showing a correlation between diastolic blood pressure and mean arterial pressure with rs217727 polymorphism [136]. Two other H19 polymorphisms, rs2107425 and rs2251375, however, were not associated with the age onset or recurrence of IS among 657 stroke patients from a Chinese population [137]. In contrast with the polymorphism studies in CAD, where several SNPs on H19 were shown to associate with reduced risk [71,121], none of these IS-associated SNPs are indicative of a decreased risk, which may be suggestive of a tissue-specific function of H19.

Other association studies look into the circulating levels of H19 and its potential as biomarker. The level of H19 in the circulation has been reported to increase in patients with IS and are positively correlated with the National Institute of Health Stroke Scale Scores within 3 h after stroke onset and 7 days after thrombolytic treatment [83]. The correlation between circulating H19 levels and the neurological deficit scores in these patients are still observed after 7, 30, and 90 days post cerebral ischemic attack [85], suggesting a high diagnostic and prognostic value for circulating H19 in the context of IS [83,85]. Nevertheless, as these studies are associative, no conclusion can be derived on whether increased H19 expression is causative or consequential. The increased H19 levels in the blood may originate from the brain or other affected tissues following the ischemic insult, as a result of an inflammatory response, cellular damage, disruption of the blood–brain barrier and release of the cell content to the circulation [83]. Loss- or gain-of-function studies are needed to address this question.

#### The seemingly controversial role of H19 in IS

H19 inhibition in middle cerebral artery occlusion (MCAO) model in mice has been shown to accelerate recovery of neurological deficits [85]. Likewise, another study reported enhanced axonal sprouting in the spinal cord and improved recovery of neural function following H19 knockdown in a MCAO mouse model [86]. Excessive neuroinflammation has been shown to induce brain damage upon stroke [138]. Interestingly, loss of H19 at the onset of ischemia in a rat MCAO model prevented the induction of TNF-α, IL-1β and IL-6 concentrations in plasma and brain tissue within 24 h after stroke onset [83]. H19 silencing was shown to reduce infarct volume and brain edema in the acute stage and rescued the loss of brain tissue and neurological deficits in the recovery stage [83,85]. It was shown that microglia, the brain-resident immune cells [139], were the responsible cell type with increased H19 expression, which induces the I/R damage by promoting an excessive inflammatory response [83]. The loss of H19 was shown to reduce histone deacetylase 1 (HDAC1) and increased acetyl-histone H3 and H4 during oxygen-glucose deprivation/re-oxygenation (OGD/R), which promotes microglial polarization from the M1 to the M2 phenotype, hence dampening its inflammatory response and alleviating the tissue damage (Figure 3D) [83]. In line, H19 was identified as the most up-regulated lncRNA upon I/R injury of the microglia in the retina, the surrogate peripheral tissue of the brain. It is proposed that H19 can form a ceRNA network with miR-21 and PDCD4 to regulate the NLRP3/6 inflammasome. I/R-induced up-regulation of H19 causes an imbalance in the NLRP3/6 inflammasome that leads to a proinflammatory cytokine response, microglial pyroptosis, and ultimately neuronal apoptosis as a consequence of an excessive immune response (Figure 3D) [87].

Other mechanistic insights have been derived from an OGD/R model in other cell types than microglia, including neuroblastoma and immortalized hypocampal cells. A study suggests that H19 can modulate PTEN/AKT3 signaling by acting as a ceRNA for miR-19a-3p in neuroblastoma cells, thus aggravating cerebral I/R injury [88]. Similarly, H19 was shown to bind miR-29b to regulate SIRT1 and peroxisome proliferator-activated receptor-gamma coactivator-1α (PGC-1α) expression in an immortalized mouse hippocampal cell line [89]. Further, administration of metformin during I/R injury in Neuro2a cells, another neuroblastoma cell line, was shown to reduce the expression of H19 and Rho-associated protein kinase 2 (Rock2), which protects from cellular damage [90]. Nevertheless, as these model cells are immortalized cell lines, they may not be representative to the physiological situation *in vivo*. Hence, the mechanistic insights derived from these cells do not necessarily reflect what happens in the body. In contrast, the microglia appears to be more relevant given their presence in the brain. Increased H19 levels in microglia appear to activate and enable the cells to perform their function as inflammatory cells in order to clear the cellular debris upon ischemic insult. However, even though the intention is good, continuous microglia activation leads to an excessive inflammatory response that eventually damages the brain. Hence, inhibiting H19 in the microglia can be therapeutic against IS.

On the other hand, in contrast to the proposed neuroprotective effect of H19 silencing, another study suggests that H19 acts as mediator of stroke-induced neurogenesis [91]. Here, H19 was identified as most up-regulated lncRNA in neural stem cells (NSCs) of the subventricular zone from rats following focal cerebral ischemia. Interestingly, using CRISPR-Cas9 genome editing, knocking out H19 in NSCs inhibited cellular proliferation as well as neuronal differentiation and survival, resulting in worsening of motor and cognitive deficits post-ischemia. Mechanistically, H19 recruits chromatin remodeling PRC2 core subunits SUZ12 and enhancer of zeste homolog 2 (EZH2) to promote repressive H3K27 trimethylation on major cell cycle- and apoptosis-related genes, including p21, PTEN and p27 (Figure 3D) [91]. Moreover, silencing of H19 in ischemic NSCs not only down-regulated miR-675 but also suppressed the biogenesis of neurogenesis-related miRNAs (miR-146a, miR-17-92 cluster, miR-21, miR-124a, and miR23), possibly through a reduced Dicer expression [91,140].

Whether the loss of H19 is protective or detrimental, it seems to depend on which neuronal cell type is targeted by the silencing agent. The studies showing the protective effect of H19 silencing used a rather systemic approach by administering the siRNA into the brain ventricle [83,85]. This approach may allow the uptake of the siRNA primarily by microglia as the most inflammatory active cells patrolling the brain upon injury. In contrast, the study showing the detrimental role of H19 silencing appears to use a more targeting approach. It was shown that in order to silence H19 in NSCs, the silencing agent was administered directly in the subventricular zone, the area of the brain where the NSCs are located [91]. This way, the primary cell type incorporating the treatment are the NSCs, and as H19 up-regulation in NSCs upon I/R injury serves to enhance proliferation of this cell type, its silencing leads to cellular damage and worsening of the brain injury.

#### Neuroprotective effect of H19 in hypoxic-ischemic encephalopathy

In the context of hypoxic–ischemic encephalopathy (HIE), a major cause of neurological disabilities among newborns due to perinatal asphyxia [141], H19 overexpression was shown to protect against brain damage [92]. Although H19 levels were up-regulated in brain tissue of neonatal HIE rats, it appears to serve as a beneficial, neuroprotective response rather than causing detrimental effects following brain ischemia [92]. On a molecular level, H19-mediated neuroprotection is achieved by sponging miR-29b, leading to an up-regulation of the AKT3/mTOR pathway and consequently blocking excessive autophagy in the brain [92]. Another study found that H19 expression levels decreased in neurons from a hypoxic–ischemic brain damage (HIBD) neonatal rat model, while overexpression of H19 showed a similar protective effect, alleviating cognitive dysfunction and neuronal apoptosis upon HIBD [93]. In primary neuron cells from neonatal rats, it was shown that H19 competitively binds to miR-107, thereby increasing the expression level of vascular endothelial growth factor (VEGF) (Figure 3D) [93], which is known for its neuroprotective effect by inducing neurogenesis and cerebral angiogenesis following HIBD [142]. Consistent with this, H19 overexpression in primary embryonal rat NSCs was shown to activate Wnt/β-catenin and PI3K/AKT3 pathways by regulating miR-107 expression *in vitro*, leading to attenuation of hypoxia-induced injury [94].

#### The role of H19 in haemorrhagic stroke (HS)

Although IS accounts for the majority of stroke cases, HS is associated with the highest mortality rate in patients [143]. The role of H19 in HS has not yet extensively been studied as compared with IS. H19 was identified as the most up-regulated lncRNA in the brain 24 h after the induction of intracerebral hemorrhage (ICH) in two distinct rat models and its expression remained elevated for 7 days after ICH. Although the molecular mechanism was not fully elucidated, *in silico* gene and protein interaction analysis revealed that high H19 expression levels following ICH were associated with the type I interferon signaling pathway [95].

In another HS subtype, namely subarachnoid hemorrhage (SAH), which is characterized by a bleeding near the brain surface [144], H19 expression was found down-regulated [96]. In an attempt to study the protective effect of melatonin against early brain injury following SAH in mice, it was demonstrated that H19 expression is induced by melatonin in a concentration-dependent manner [96]. Melatonin treatment decreased cell senescence and apoptosis in the brain cortex, resulting in improved neurological deficits and reduced brain edema after SAH [96]. On a molecular level, up-regulation of H19 by melatonin increased the expression of miR-675, while decreasing the expression of its competing miRNA let-7a. As miR-675 and let-7a target p53 and neural growth factor (NGF) (Figure 3D), respectively, the administration of melatonin reduced p53 and enhanced NGF levels [96]. One of the leading complications of SAH is cerebral vasospasm, which implies the narrowing of major cerebral arteries [145]. Consistent with previous findings [96], melatonin treatment was shown to rescue the SAH-induced vasospasm and neurological deficits by increasing the expression of H19 in the brain [97]. It was demonstrated that H19 competitively binds to miR-138 to de-repress the expression of endothelial nitric oxide synthase (eNOS), whereas melatonin-induced expression of miR-675 inhibits SAH-mediated accumulation of hypoxia-inducible factor 1α (HIF-1α) and its target VEGF [97], leading to attenuation of SAH-induced vasospasm and neurobehavioral deficits [97].

Overall, these studies highlight again the pleiotropic properties of H19 in a cell-specific context. Increased levels of H19 appear to serve as a protective response in various brain cell types. However, prolonged expression, such as in microglia, can induce an excessive immune response, leading to neuronal damage. In other cell types, such as NSCs and primary neuronal cells, H19 enhances cell viability and prevents apoptosis, which improves neural function. Therefore, silencing or overexpression of H19 may be detrimental or beneficial depending on which cell type is primarily targeted by the treatment. Hence, specific delivery methods and timing of administration are key factors to consider in order to target the culprit cell type, while harnessing the beneficial effect of H19 in the brain.

### H19 in aneurysmal disease

Aneurysms, a ballooning in a blood vessel caused by a weakening of the vessel wall, can contribute to internal hemorrhage, leading to life-threatening and devastating situations. Infiltration of immune cells and phenotypic switching of VSMCs appear to play a crucial role in the pathophysiology of aneurysm formation [146]. Phenotypic switching of VSMCs from a contractile to a synthetic phenotype is characterized by an increased proliferative capacity, decreased levels of contractile protein, and increased production of elastolytic enzymes, which degrade the extracellular matrix (ECM) and facilitate cell migration [147]. Polymorphisms on the H19 genomic locus have been associated with increased risk of developing intracranial aneurysms [148]. H19 was found up-regulated in the intracranial aneurysm tissue as compared with the adjacent normal arterial tissue from the same patient [98]. In addition to intracranial aneurysm, H19 levels were up-regulated in aneurysm biopsies taken from patients with abdominal aortic aneurysm (AAA) as compared with the abdominal aortic tissue from healthy controls [99]. Similarly, H19 levels were higher in the AAA samples in comparison with the adjacent normal aortic tissue from the same patient [100]. H19 levels were also higher in two pre-clinical models of AAA, namely angiotensin II-infused ApoE^−/−^ mice [99,100] and LDLR^−/−^ Yucatan mini-pigs [99]. Moreover, increased expression of H19 was evident in serum and thoracic aortic aneurysm (TAA) tissue from patients with TAA as compared with non-TAA controls [101].

The mechanistic insights on how H19 regulates VSMCs leading to aneurysm formation seem conflicting. On the one hand, it was shown that overexpression of H19 induces human aortic SMC apoptosis and inhibits their proliferation, an effect that was independent of miR-675. Nuclear H19 appears to recruit transcription factor specificity factor 1 (Sp1) to the promoter region of HIF-1α, which enhances its expression. Increased cytoplasmic HIF-1α interacts with Mouse double minute 2 homolog (MDM2) and prevents MDM2-mediated reduction of p53, leading to elevated BAX and decreased BCL2 levels, which trigger SMC apoptosis and accelerate AAA development (Figure 3E) [99]. Moreover, it was reported that H19 overexpression in VSMCs inhibited proliferation, promoted ECM degradation, and induced apoptosis by acting as a ceRNA to miR-1-3p, leading to the de-repression of a miR-1-3p target gene, an elastolytic protein disintegrin-like metalloproteinase ADAM10 [101].

On the other hand, another study showed that H19 induced VSMC proliferation [102]. It was reported that H19 sponged miR-193-3p to induce a switch of human aortic VSMC from a contractile to a synthetic phenotype with higher proliferation, migration and synthesis capability, but lower capacity to maintain vascular tone (Figure 3E) [102]. This study is in line with the previously mentioned studies on the role of H19 in atherosclerotic plaques, where H19 also promotes VSMC proliferation and inhibits cell apoptosis [55,56,58,64]. In addition, H19 was shown to induce AAA development primarily by promoting inflammation via intensifying aortic IL-6 and MCP-1 levels and macrophage infiltration, and not through the regulation of VSMC proliferation. H19 was proposed to act as an endogenous competitor for let-7a miRNA to induce the transcription of its target gene IL-6 in mouse aortic VSMCs and macrophages (Figure 3E) [100].

It is not clear why H19 seems to have different effects on VSMC proliferation. All studies appear to use human aortic SMCs in their *in vitro* model. However, the developmental origin of the SMCs, whether it is thoracal or abdominal, may render different responses to H19 [146,149]. Another explanation would be technical differences in the culturing and transfection procedure, which are not immediately apparent from the literature. Nevertheless, it seems more likely that upon injury, such as angiotensin II stimulation or CaCl_2_ exposure, VSMCs respond by increasing its proliferative capacity under the regulation of H19, as a mean to protect the vessel wall integrity. However, in doing so, VSMCs secrete elastolytic enzymes and lose their ability to regulate vasomotor tone, which in the long term leads to weakening and bulging of the vessel wall. Similarly in macrophages, increased H19 may serve as a protection to enable these cells to clear up damage upon injury. However, an extensive proinflammatory response can eventually induce damage on the VSMCs and their surrounding tissue, leading to weakening of the vessel wall [146].

### H19 in pulmonary hypertension

Pulmonary arterial hypertension (PAH) is a vascular remodeling disease characterized by vasoconstriction and progressive obliteration of distal pulmonary arteries leading to elevation of pulmonary pressure and eventually right ventricular (RV) HF [150]. Pulmonary vascular remodeling is characterized by the excessive proliferation of pulmonary arterial smooth muscle cells (PASMCs) and dysfunction of pulmonary arterial endothelial cells [151].

The level of H19 in plasma has been reported to increase in patients with PAH, whereby circulating H19 levels showed a negative correlation with RV function and can be used to predict long-term outcome in two independent PAH cohorts [103]. Further, H19 was up-regulated in the decompensated RV from PAH patients and its level positively correlates with RV hypertrophy and fibrosis [103]. In addition, it is increased in the lungs and RV of two models of PAH, namely monocrotaline (MCT) injection [103,104] and pulmonary artery banding (PAB) in rodents [103]. This is in contrast with the pressure overload-induced left ventricular failure, where H19 expression at the decompensated stage was down-regulated [79], suggesting different roles of H19 in the left versus right part of the heart. Silencing of H19 limits pathological RV hypertrophy, fibrosis, and capillary rarefaction, thus preserving RV function in MCT and PAB rats [103,104]. However, the author did not seem to study the effect of H19 silencing in the PASMCs or endothelial cells, which are particularly more relevant in the MCT model, as it has been shown to induce dysfunction in these cells, leading to PAH [152,153]. As the silencing agent was administered systematically by intraperitoneal injection, it is plausible that normalization of the pulmonary artery contribute to the protective effect H19 silencing. Mechanistically, this cardioprotective effect was accompanied by E2F transcription factor 1 (E2F1)-mediated up-regulation of EZH2 in CMs (Figure 3F) [103]. Moreover, the authors indicate that cardiac fibroblasts also played a crucial role in the progression of RV failure as silencing of H19 prevents the differentiation of these cells into myofibroblasts [103]. Nevertheless, as this result was inferred from an *in vitro* model, it needs to be verified *in vivo* by assessing the differential expression of H19 in different cardiac cell types of the RV after PAH injury, ideally including loss-of-function experiments with gapmeR treatment.

Another study showed increased H19 levels in the lungs and PASMC exposed to MCT. It was proposed that H19 up-regulated angiotensin II type 1 receptor (AT1R) expression via sponging let-7b, thereby promoting PASMCs proliferation, *in vitro* (Figure 3F) [104]. This is in line with other studies on H19 in atherosclerosis and aneurysm where H19 induces VSMC proliferation [55,56,58,64,102]. On the other hand, it was shown that H19 expression was reduced in the PASMC extracted from rats subjected to MCT [105]. Here, increased H19 levels were shown to mediate the therapeutic effect of melatonin in this PAH rat model by sponging miR-675-3p, thereby releasing its inhibition on IGF1R, and by inhibiting miR-200a, leading to the de-repression of PDCD4, which promoted apoptosis and suppressed proliferation of human and rat PASMCs, *in vitro* (Figure 3F) [105]. This study seems in contrast with other studies showing H19-induced VSMC proliferation. Nevertheless, this study did not incorporate loss- or gain-of-function experiments for H19. Therefore, it cannot be excluded that the reduction of PASMC proliferation is the effect of melatonin *via* other downstream pathways, and not necessarily through the H19 axis.

## Conclusion and clinical perspectives

Over the past decades, the pleiotropic lncRNA H19 has been attracting widespread interest as an oncofetal RNA gene acting on multiple layers of gene expression regulation. A growing body of literature has now started to shed light on the importance of H19 in cardiovascular homeostasis and diseases, making this lncRNA an attractive candidate as biomarker and target therapy. However, the mode of actions of H19 in vascular diseases are still at its infancy and remain to be fully elucidated. Many studies focusing on H19 dysregulation are limited to *in vitro* observations and therefore may not accurately reflect the complex interplay of spatiotemporal expression patterns in a cell- and tissue-specific environment *in vivo*. In addition, numerous studies propose that H19 acts as a ceRNA to exert its biological function. However, the impact of its role as miRNA sponge in vascular diseases needs to be carefully considered. For example, the let-7 family of miRNAs is highly abundant in vascular cells as compared with the sparse expression of H19, questioning on how H19 competes with this high number of miRNAs to mediate its downstream effect [154]. Thus, it is first and foremost essential to better understand the diverse and partly contrasting mechanisms of action of H19 before considering the clinical translation of these studies. The discrepancies on the effect of H19, whether it is protective of detrimental, may be explained by its cell type-specific function. In VSMCs, H19 seems to induce phenotypic switching to a synthetic, non-contractile form with an active proliferative state, while in immune cells such as macrophages, it induces M1 polarization and enhances the cell immune response. Even though this response is protective to maintain vessel wall integrity and to clear up cellular debris upon insult, overexpressing H19 in these cells may be detrimental as prolonged VSMC switch to a synthetic phenotype may contribute to the formation of atherosclerotic plaque and aneurysm, whereas M1 polarization may induce an excessive proinflammatory response that is damaging the surrounding tissue.

Nevertheless, the development of H19-targeted therapies in the context of cardiac hypertrophy has recently sparked considerable attention [155]. Intriguingly, cardiac-specific overexpression of H19 via vector-based gene therapy ameliorated heart function in a mouse model of afterload-induced HF, even when H19 delivery occurred after cardiac hypertrophy had been established [79]. Of note, cell type-specific targeting of H19 silencing or overexpression should be of an important consideration for the therapeutic use of this lncRNA. This can be achieved by dose adjustment [118,119] or by using a specific delivery vector for the targeted cells [124,156,157]. Moreover, time of administration is of particular importance, since overexpressing H19 during the compensatory period in certain cell types may prove detrimental, and therefore, silencing approaches are more desirable at this particular period. Further, the potential value of H19 as diagnostic and/or prognostic biomarker has also been extensively discussed. Detection of H19 gene polymorphisms or aberrant levels of circulating H19 may deliver valuable diagnostic information when used as a routine healthcare measurement, although this might only be true for specific subpopulations. Yet, exploiting the diagnostic and therapeutic potential of lncRNAs such as H19 in cardiovascular diseases could pave the way for combatting one of the greatest healthcare burdens worldwide.

## Abbreviations

α-SMA: α-smooth muscle actin
AAA: abdominal aortic aneurysm
ACP5: acid phosphatase 5
Ang II: angiotensin II
ANGPTL4: angiopoietin-like 4
ApoE^−/−^: apolipoprotein E-deficient
AT1R: angiotensin II type 1 receptor
BAX: BCL2-associated X
BCL2: B-cell lymphoma
CAD: coronary artery disease
ceRNA: competing endogenous RNA
CM: cardiomyocyte
CVD: cardiovascular disease
DR: diabetic retinopathy
E2F1: E2F transcription factor 1
ECM: extracellular matrix
EC: endothelial cell
ELF5: E74-like factor 5
EndoMT: endothelial-to-mesenchymal transition
eNOS: endothelial nitric oxide synthase
ERK: extracellular-signal-regulated kinase
EU: European Union
EZH2: enhancer of zeste homolog 2
FSP-1: fibroblast-specific protein 1
HDAC1: histone deacetylase 1
HF: heart failure
HFD: high-fat diet
HIBD: hypoxic-ischemic brain damage
HIE: hypoxic-ischemic encephalopathy
HIF-1α: hypoxia-inducible factor 1α
HS: haemorrhagic stroke
HUVEC: human umbilical vein endothelial cell
I/R: ischemia reperfusion
ICH: intracerebral hemorrhage
IGF: insulin­-like growth factor
IL: interleukin
iPSC: inducible pluripotent stem cell
IS: ischemic stroke
LAD: left anterior descending
lncRNA: long non-coding RNA
MAPK: mitogen activated protein kinase
MCAO: middle cerebral artery occlusion
MCT: monocrotaline
MDM2: mouse double minute 2
MI: myocardial infarction
ncRNA: non-coding RNA
NF-κB: nuclear factor-kappa B
NFAT: nuclear factor of activated T cells
NGF: neural growth factor
NSC: neural stem cell
OGD/R: oxygen-glucose deprivation/re-oxygenation
ox-LDL: oxidized low-density lipoprotein
PAB: pulmonary arterial banding
PAH: pulmonary arterial hypertension
PAPPA: pappalysin 1
PASMC: pulmonary arterial smooth muscle cell
PGC-1α: peroxisome proliferator-activated receptor-gamma coactivator-1α
PRC2: polycomb repressive complex 2
RIP: RNA immunoprecipitation
Rock2: Rho-associated protein kinase 2
RV: right ventricular
SAH: subarachnoid hemorrhage
sEV: small extracellular vesicle
SIRT1: sirtuin-1
SNP: small-nucleotide polymorphism
Sp1: specificity factor 1
STAT3: signal transducer and activator of transcription 3
TAA: thoracic aortic aneurysm
TAC: transverse aortic constriction
TGF: transforming growth factor
TNF: tumor necrosis factor
VE: vascular endothelial
VEGF: vascular endothelial growth factor
VSMC: vascular smooth muscle cell
XBP1: X-box-binding protein 1
YB: Y-box-binding protein
